# Characterizing QT interval prolongation in early clinical development: a case study with methadone

**DOI:** 10.1002/prp2.284

**Published:** 2017-01-24

**Authors:** Vincent F. S. Dubois, Meindert Danhof, Oscar Della Pasqua

**Affiliations:** ^1^ Division of PharmacologyLeiden Academic Centre for Drug ResearchLeiden UniversityLeidenthe Netherlands; ^2^ Clinical Pharmacology Modelling & Simulation, GlaxoSmithKlineStockley ParkUxbridgeUnited Kingdom; ^3^ Clinical Pharmacology & TherapeuticsUniversity College LondonLondonUnited Kingdom

**Keywords:** Clinical trial simulations, methadone, PKPD modeling, QT interval prolongation, translational pharmacology.

## Abstract

Recently, we have shown how pharmacokinetic–pharmacodynamic (PKPD) modeling can be used to assess the probability of QT interval prolongation both in dogs and humans. A correlation between species has been identified for a drug‐specific parameter, making it possible to prospectively evaluate nonclinical signals. Here, we illustrate how nonclinical data on methadone can be used to support the evaluation of dromotropic drug effects in humans. ECG and drug concentration data from a safety pharmacology study in dogs were analyzed using nonlinear mixed effects modeling. The slope of the PKPD model describing the probability of QT interval prolongation was extrapolated from dogs to humans and subsequently combined with methadone pharmacokinetic data as input for clinical trial simulations. Concentration versus time profiles were simulated for doses between 5 and 500 mg. Predicted peak concentrations in humans were then used as reference value to assess the probability of an increase in QT interval of ≥5 and ≥10 ms. Point estimates for the slope in dogs suggested low probability of ≥10 ms prolongation in humans, whereas an effect of approximately 5 ms increase is predicted when accounting for the 90% credible intervals of the drug‐specific parameter in dogs. Interspecies differences in drug disposition appear to explain the discrepancies between predicted and observed QT prolonging effects in humans. Extrapolation of the effects of racemic compound may not be sufficient to describe the increase in QT interval observed after administration of methadone to patients. Assessment of the contribution of enantioselective metabolism and active metabolites is critical.

**Abbreviations**
CYPs: cytochrome P450s
FTIH: first time in human
GLP: Good Laboratory Practice
NMDA: N‐methyl‐D‐aspartate
PKPD: pharmacokinetic–pharmacodynamic

## Introduction

Drug‐related prolongation of the QT interval has been a cause of attrition and concern during the screening and selection of candidate molecules (Shah 2007). Despite the availability of multiple screening methods, experimental protocols continue to be applied from early discovery without clear understanding whether drug effects in these experiments predict clinically relevant QT prolongation when administered to humans at therapeutic doses. As a consequence, uncertainty about the liability for QT prolongation has been managed by estimating the effect size in the so‐called thorough QT study (TQT), which has been a regulatory requirement for all drugs in development for more than a decade.

Whereas alternative approaches have been recently proposed, which shift the focus of screening procedures toward the evaluation of proarrhythmic rather than dromotropic properties of compounds during preclinical development, such a liability may not be fully dismissed without further characterization of the underlying concentration–effect relationship. In fact, this represents an important caveat of the methodology for the estimation of QT interval prolongation which has been recommended over the last decade (FDA, 2005). The use of the so‐called double‐delta method has been shown to cause high false‐positive rates and most importantly to ignore the underlying concentration–effect relationship, given that statistical significance is assessed at the dose/treatment level (France and Della Pasqua 2015). These limitations have prompted scientists and regulators to endorse and consider careful evaluation of pharmacokinetic–pharmacodynamic (PKPD) relationships during the analysis of TQT study data. However, the whole approach presents considerable limitations, as it does not enable conclusive decisions to be taken about the development of a new compound before Phase IIb or III. This situation has led to the recent proposal to include appropriate QT assessments in an earlier stage of the clinical drug development. More specifically to characterize drug‐induced effects on QT interval during first‐time‐in‐humans studies, during which single and multiple ascending doses are evaluated. PKPD modeling can then be used as a tool to assess the extent of QT/QTc prolongation in a strictly quantitative manner (Darpo et al. 2014). These data should be complemented by results from a comprehensive in vitro proarrhythmia assay (CiPA) (Cavero and Holzgrefe 2015; Sager et al. 2014; Fermini et al. 2016).

It should be highlighted that the premise for the ongoing review of the guidelines is that QT interval prolongation is not a specific surrogate marker for the risk of torsades de pointes. In fact, electrophysiological studies have provided insight into the mechanisms of drug‐related arrhythmogenicity. Such studies are presently pursued within the CiPA initiative (Fermini et al. 2016). It can be anticipated that novel biomarkers will be identified which ultimately can be of value for use as surrogate markers. Meanwhile in the absence of specific markers, the emphasis remains on the assessment of QT/QTc interval prolongation prior to the regulatory approval drugs. In addition, the approach proposed by the CiPA initiative needs to consider that differences may exist between in vitro and in vivo effects, which are not evident without an integrated analysis of the data. Therefore, in this investigation, we show how drug effects can be scaled from dog studies to humans. Ultimately the findings in these studies may prove of value in the prediction of the QT response at therapeutic doses. It can be envisaged that the tandem of CiPA and PKPD modeling may yield a strong basis for the evaluation of proarrhythmic risk.

We have recently shown that the use of a model‐based approach allows for the translation and extrapolation of preclinical findings, as exposure–response relationships can be derived using a common model parameterization (Chain and Dubois et al. 2013; Chain et al. 2013; Dubois et al. 2014, 2016a,b). In addition, the evaluation of a range of compounds showing varying degrees of hERG inhibition has revealed a correlation between drug‐specific model parameter estimates in dogs and humans, namely, the slope describing the linear relation between drug concentration and QT interval prolongation (Dubois et al. 2016a,b). Whereas we acknowledge that additional compounds with different proarrhythmic mechanisms should be integrated into the analysis to ensure higher precision of the parameter estimates, results obtained so far are robust enough to enable prospective evaluation of the clinical effects of new compounds with detectable hERG activity.

To illustrate the implementation of an improved screening procedure for new compounds, we have analyzed in a blinded manner pharmacokinetic and electrocardiographic data obtained from dogs after oral administration of methadone at dose levels typically used during in vivo safety pharmacology studies. These data were used in combination with simulated pharmacokinetic data from a hypothetical phase I dose escalation study in healthy subjects. The main goal of this investigation was to demonstrate the advantages of integrating preclinical information into clinical trial simulations to evaluate the potential effects at therapeutically relevant concentrations. Our analysis was also aimed at identifying the role of interspecies differences in drug disposition for the accurate extrapolation and prediction of proarrhythmic effects in humans. Finally attention was given to experimental design requirements, as to ensure suitable data are derived for modeling and simulation. Methadone was selected as a paradigm compound primarily because pharmacokinetic data and the corresponding effects on QT interval were available both in conscious dogs and in humans. Furthermore the effects of methadone on the QT interval are well established. Most importantly, methadone was not included in the dataset that was used to identify the interspecies correlation (Dubois et al. 2016a,b).

Methadone was first synthesized in 1939 at the pharmaceutical laboratories of I.G. Farbenconzern, a subsidiary of Farbwerke Hoechst, in Germany. One of the key objectives of the research team was to identify effective analgesic properties that would be nonaddictive. In fact, early experiments showed that the drug was both an analgesic and a spasmolytic. Only in 1947, results of both animal and human studies were presented in which doses of methadone between 200 and 800 mg given four times daily were described in relation to tolerance, physical dependence, and abstinence syndrome. The discovery of methadone's unique pharmacokinetic properties did not occur until 14 years later (Payte 1991). Today, it is well established that methadone is an opioid receptor agonist (Selley et al. 2001) and also variably acts as an N‐methyl‐D‐aspartate (NMDA) receptor antagonist and adrenergic (alpha‐2) agonist (Codd et al. 1995; Gorman et al. 1997).

Methadone is marketed as a racemic mixture and has high oral bioavailability and a long terminal half‐life (23–35 h) compared with many other opioids (Dale et al. 2002, 2004). In dogs, however, the oral bioavailability is low and terminal half‐lives range from 1.75 to 4 h and 2 to 12 h following intravenous (IV) and subcutaneous (SC) administration, respectively. Methadone's volume of distribution is large and of the same magnitude in both humans and dogs (Kukanich 2005; Kukanich and Borum 2008). The metabolic pathways of methadone have not been fully characterized in dogs, but data show that it is mainly extracted by the liver (Garrett et al. 1985) with clearance values corresponding to 89% of the hepatic blood flow in dogs (Davies and Morris 1993). This implies that oral bioavailability in this species is low. By contrast, in humans, methadone is a low‐clearance drug and the oral bioavailability is high (Meresaar et al. 1981). In addition, multiple cytochrome P450s (CYPs) have been identified in the metabolism of methadone, including CYP3A4, CYP2C8, CYP2D6, and CYP2B6 (Wang and De Vane Lindsay 2003; Totah et al. 2008).

The suspicion regarding methadone‐induced torsadogenic effects dates back to 1973 when Stimmel et al. reported prolongation of the QTc interval in narcotic drug addicted patients. Numerous publications have since then emerged describing the potential link between QTc interval prolongation and methadone use. An overview of published findings can be found elsewhere (Mujtaba et al. 2013). The first case series was reported by Krantz et al. who found an association between high‐dose methadone use (average dose of 400 mg/day) and TdP. However, no pharmacokinetic information was available and 12 of these 43 patients had hypokalemia or used another QTc prolonging medication. More recently, a dose‐dependent effect of methadone on the QTc interval has been reported, involving patients on oral methadone treatment across a dose range 10–1200 mg/day.

Given the history of its development and the current role of methadone in the therapeutic management of pain and addiction, our investigation highlights the importance of evaluating concentration–effect relationships when characterizing the safety profile of a new molecule. An important feature of the proposed modeling approach is that it enables analysis of the effects at different thresholds; that is, 10 msec (which is the upper confidence interval threshold) as required by ICH E‐14 (FDA 2005), but also 5 msec (which is the threshold of the average prolongation). This is particularly interesting for drugs with a borderline effect such as methadone or for those which show high propensity for pharmacokinetic drug–drug interactions. Using clinical trial simulations, we show how such data can be used in combination with quantitative pharmacology concepts not only to stop the development of unsuitable candidate molecules but also to understand liabilities and mitigate risks during clinical development.

## Material and Methods

### Dog study

Conscious male beagle dogs (*n* = 4) were given single subcutaneous doses of vehicle (0.9% (w/v) sodium chloride pH 4.5+/− 0.1 adjusted) and methadone (Batch Number, 028K1166, 89.5% pure as methadone, assigned retest date of 22 January 2011) at 0.2, 0.6, and 2 mg/kg. Arterial blood pressure, heart rate, electrocardiographic intervals, QA interval (index of cardiac contractility), and body temperature were monitored for 24 h after dosing. Electrocardiographic waveforms were evaluated for disturbances in waveform rhythm and morphology following treatment with vehicle and methadone at all doses. Blood samples were taken for measurement of blood concentrations of methadone at 1, 2, 4, 8, and 24 h postdose. All animals had at least a 26‐week wash‐out period between dose levels and underwent a health check by a site veterinarian prior to the start of the study. On the first day of treatment the dogs were approximately 1.8–3.9 years old, and in the weight range 13.03–15.15 kg. Animals received injection volumes of 0.5 mL/kg, administered subcutaneously; the site of injection was changed every week. The dose volume was calculated on the day of dosing from the individual body weight (recorded weekly). Food was withheld for approximately 21 h prior to each treatment, and on treatment days, food was offered 1 h after dosing.

Methadone hydrochloride (Batch no. 028K1166, 89.5% pure as methadone) was supplied by Sigma and was stored at the appropriate storage conditions at room temperature, protected from light. Solutions at concentrations of 0, 0.4, 1.2, and 4 mg/mL methadone in 0.9% (w/v) aqueous sodium chloride, pH 4.5 adjusted, were prepared shortly prior to dosing as no stability information was available for methadone hydrochloride in this vehicle.

All experiments were approved by the ethical committees and performed under Good Laboratory Practice (GLP) regulations. The animals were housed singly and the environmental controls were set to maintain temperature at 19 ± 2°C in the holding bay in which there was a 12‐h light/12‐h dark cycle. Dogs were fed Harlan Teklad 2021C Dog Maintenance diet except when treated and provided with Datesand Grade 6 bedding. Filtered water was available ad libitum. Summary study details are provided in Table 1. Details of the bioanalysis and subsequent modeling of the data can be found in the supplemental material.

**Table 1:** Experimental protocol design and sampling times in dogs

Number of animals	4
Gender	M
Weight [kg]	14 (13–15.5)
Dose [mg/kg]	0, 0.2, 0.6, 2.0
PK sampling times [h]	0, 1, 2, 4, 8, 24
PD sampling times	Every 30 sec, averages over 24 h
PK parameter	Plasma concentration
Vital signs	Heart rate, blood pressure
Demographic covariates	Weight, sex
ECG parameters	MBP, SBP, DBP, PP, PR, QT, HR, RR, QRS, Temp

DBP, diastolic blood pressure; HR, heart rate; MBP, mean blood pressure; SBP, systolic blood pressure; Temp, body temperature.

### Prediction of QT prolongation in humans

In order to accurately simulate the putative drug‐induced QT‐prolonging effects in a clinical trial in human subjects, two different scenarios were considered: (1) A randomized, placebo‐controlled, crossover, dose‐escalation study, similar to a typical first‐time‐in‐human (FTIH) protocol with a small group of subjects (*N* = 27), in which a range of doses are evaluated in an escalating manner in conjunction with an active comparator; and (2) a thorough QT study (TQT) in which a larger group of subjects (*N* = 60) are administered no more than two or three dose levels according to a parallel and a crossover design.

To evaluate such scenarios, pharmacokinetic data have to be simulated for all subjects using a preliminary PK model from single doses of the compound. These data reflect typical single ascending dose pharmacokinetic protocols in phase I trials. As no detailed information about drug metabolism in humans is available at this stage of development, it was assumed that active metabolites (i.e., with potential proarrhythmic effects) are not formed. The individual pharmacokinetic profile in humans was then simulated 150 times for each of the scenarios highlighted above. For a full overview of the different study designs see Table 2.

**Table 2:** Design characteristics of the FTIH and TQT studies used in the simulations of drug‐induced QT effects in humans

Study design Scenario	FTIH crossover	TQT crossover	TQT crossover with time‐matched baseline	TQT parallel design with time‐matched baseline
Treatment (dose levels)	5 dose levels, 3 consecutive escalating doses, placebo controlled, and an active control	1 dose (top dose from FTIH), placebo controlled, and an active control	1 dose (top dose from FTIH), placebo controlled, and an active control	1 dose (top dose from FTIH), placebo controlled, and an active control
Sample size	*N* = 27	*N* = 60	*N* = 60	*N* = 60
Baseline QT	Baseline QT sampled 3 h before dosing	Baseline QT sampled 3 h before dosing	Time‐matched baseline QT sampled on the day before dosing	Time‐matched baseline QT sampled on the day before dosing
Randomization	3 cohorts: Cohort 1: placebo, 40, 80, and 120 mg methadone Cohort 2: placebo, 120, 160 and 200 mg methadone Cohort 3: placebo, 200, 250, and 500 mg methadone	2 cohorts: Cohort 1: first 500 mg methadone, then placebo and an active control Cohort 2: first placebo, then 500 mg methadone and an active control	2 cohorts: Cohort 1: first 500 mg methadone, then placebo and an active control Cohort 2: first placebo, then 500 mg methadone and an active control	2 cohorts: Cohort 1: placebo Cohort 2: first placebo, then 500 mg methadone Cohort 3: first placebo then an active control
Threshold for the probability of QT interval prolongation	5, 10 ms	10 ms	5, 10 ms	10 ms

FTIH, First‐time‐in‐human; TQT, thorough QT.

To ensure realistic levels of variability in the simulation of drug effects on QT interval, RR profiles had to be generated for each subject. This was performed using a large pool of ECG data from healthy subjects (*n* = 776, 339 males and 437 females) with an average age of 31.1 (SD 9.4) years. The starting RR interval (RR_0_) used in the simulations was based on the pooled data. This procedure was aimed at mimicking variability and dispersion in real subjects. Details of the RR simulation method can be found in Bellanti et al. (2011). A frequent sampling scheme was used to generate RR intervals (Table 3). The selected sampling times are in line with safety monitoring procedures in a typical Phase I protocol.

**Table 3:** Population PKPD parameter estimates obtained from the simulation of methadone‐induced QT‐prolonging effects in FTIH and TQT studies.^1^

Parameter	FTIH average 10 ms	FTIH average 5 ms	FTIH WCS 10 ms	FTIH WCS 5 ms	TQT crossover time‐matched BL average 10 ms	TQT crossover time‐matched BL average 5 ms	TQT crossover time‐matched BL WCS 10 ms	TQT crossover time‐matched BLWCS 5 ms
Slopem (ms/nM)	6.13E‐05	6.30E‐05	6.61E‐04	6.61E‐04	2.62E‐04	2.62E‐04	6.70E‐04	6.89E‐04
Slopef (ms/nM)	6.37E‐05	6.55E‐05	6.86E‐04	6.86E‐04	2.72E‐04	2.72E‐04	6.96E‐04	7.15E‐04
Alpha	0.338	0.338	0.338	0.338	0.339	0.339	0.338	0.338
Amplitude (ms)	4.20	4.20	4.22	4.22	3.13	3.13	3.13	3.18
Phase (h)	9.93	9.93	9.92	9.92	10.38	10.38	10.37	10.35
QTc0m (ms)	387.3	387.3	387.3	387.3	387.4	387.4	387.4	387.4
QTc0f (ms)	402.3	402.3	402.3	402.3	402.3	402.3	402.3	402.3

Simulated data were analysed using the PKPD model developed previously by Chain et al (2011). Predicted drug concentrations in humans and RR data were used in conjunction with extrapolated estimates of the slope parameter observed in dogs. The interspecies correlation for this drug‐specific parameter is based on the assumption that QT prolongation is determined by hERG inhibition mechanisms. BL, baseline; FTIH, First‐time‐in‐human; TQT, thorough QT; WCS, worst case scenario.

^1^An overview of all PKPD parameter estimates including alternative scenarios and 95% credible intervals is available in table S1 (supplemental results).

Using the predicted drug concentrations and RR intervals for each subject, QT intervals were simulated based on the PKPD model, taking into account the translational factor of 11.6 (Dubois et al. 2016a,b) (see supplemental material). The parameters describing the circadian rhythm were fixed to the values estimated previously (Dubois et al. 2016a,b), namely, 4.224 msec for the amplitude and 9.9 h for the phase. On both parameters, a 10% variability was allowed, including a residual error of 5.3 msec.

### Probability of QT interval prolongation

The model‐predicted effects of methadone are described in terms of the probability of QT prolongation above the clinical threshold of 5 and 10 msec. The Bayesian analysis was performed with a step function in WinBUGS 1.4 using the a posteriori slope estimates and an interindividual correction factor for gender differences (see Equations 1 and 2) at arbitrary concentration points between 5 and 1,00,000 nM. Data were summarized and presented in nanomolar (nM). The concentration values were chosen in such a way that data points cover the complete sigmoid curve.

(1) $$P\geq 10\text{ms (at C)}=\text{step}(0.00001^{F(\mathrm{gender})}\cdot \text{slope}\cdot \frac{5\text{ms}}{C})$$

(2) $$P\geq 10\text{ms (at C)}=\text{step}(0.00001^{F(\mathrm{gender})}\cdot \text{slope}\cdot \frac{10\text{ms}}{C})$$

where 0.00001 is set as an arbitrary small number to avoid computational errors associated with numerical difficulties (i.e., division by zero), 5 or 10 ms represent the QT interval prolongation threshold of interest, C is the drug concentration, and slope is the QT increase per unit drug concentration. Probability curves were plotted for each scenario.

## Results

### Pharmacokinetic modeling in dogs

**Figure 1:**
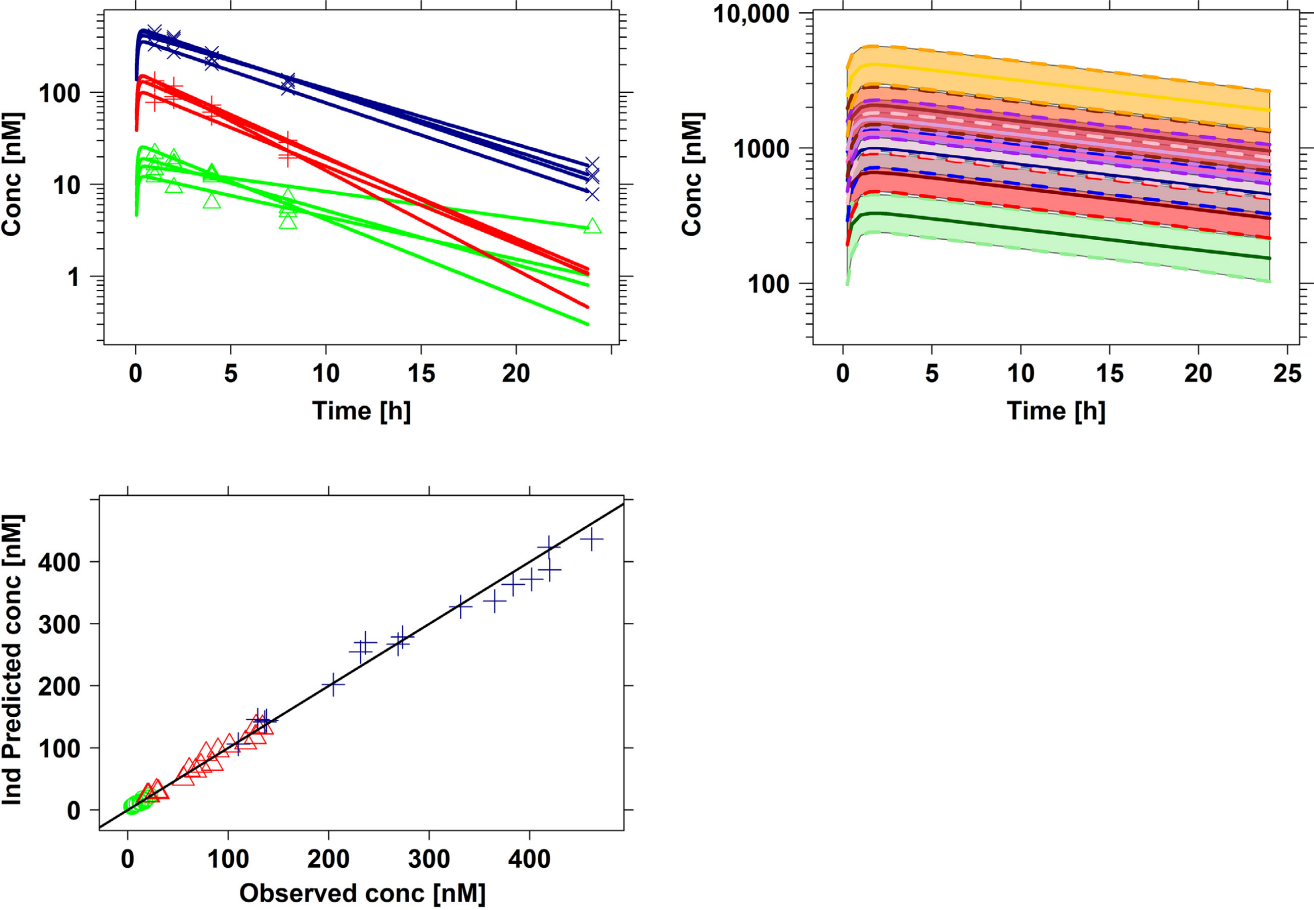
Methadone pharmacokinetics in dogs and human subjects. Concentration versus time profiles are shown in dogs (left) and in humans (right). Lines depict predicted profiles, whereas symbols indicate observed data. The experimental protocol in dogs included methadone doses of 0.2 (green), 0.6 (red), and 2 mg/kg (dark blue). Human data were simulated to mimic a cohort of 27 subjects with 7 arms, including doses of 5 (green), 10 (red), 25 (blue), 50 (pink), 100 (brown), 250 (purple), and 500 mg (orange), with IIV variability in drug disposition parameters. Note that due to species differences in pharmacokinetics, methadone exposure after administration of a 500 mg dose yields plasma levels approximately 10‐fold higher than the concentrations observed in a typical safety pharmacology protocol in dogs.

The time course of drug concentrations in dogs was analyzed in a blinded manner using nonlinear mixed effects modeling. The pharmacokinetics could be described by a one‐compartment model with oral absorption and interindividual variability on clearance and volume of distribution. The predicted profiles are depicted in Figure 1. A summary of the pharmacokinetic model and final parameter estimates is shown in Table S1 (see supplemental results). As most of the pharmacokinetic samples were taken at different time points relative to the ECG recordings, individual predicted concentrations were required to generate time‐matched data for subsequent evaluation of drug effects by PKPD modeling.

### Pharmacokinetic–pharmacodynamic modeling of the dromotropic effect in dogs

**Figure 2:**
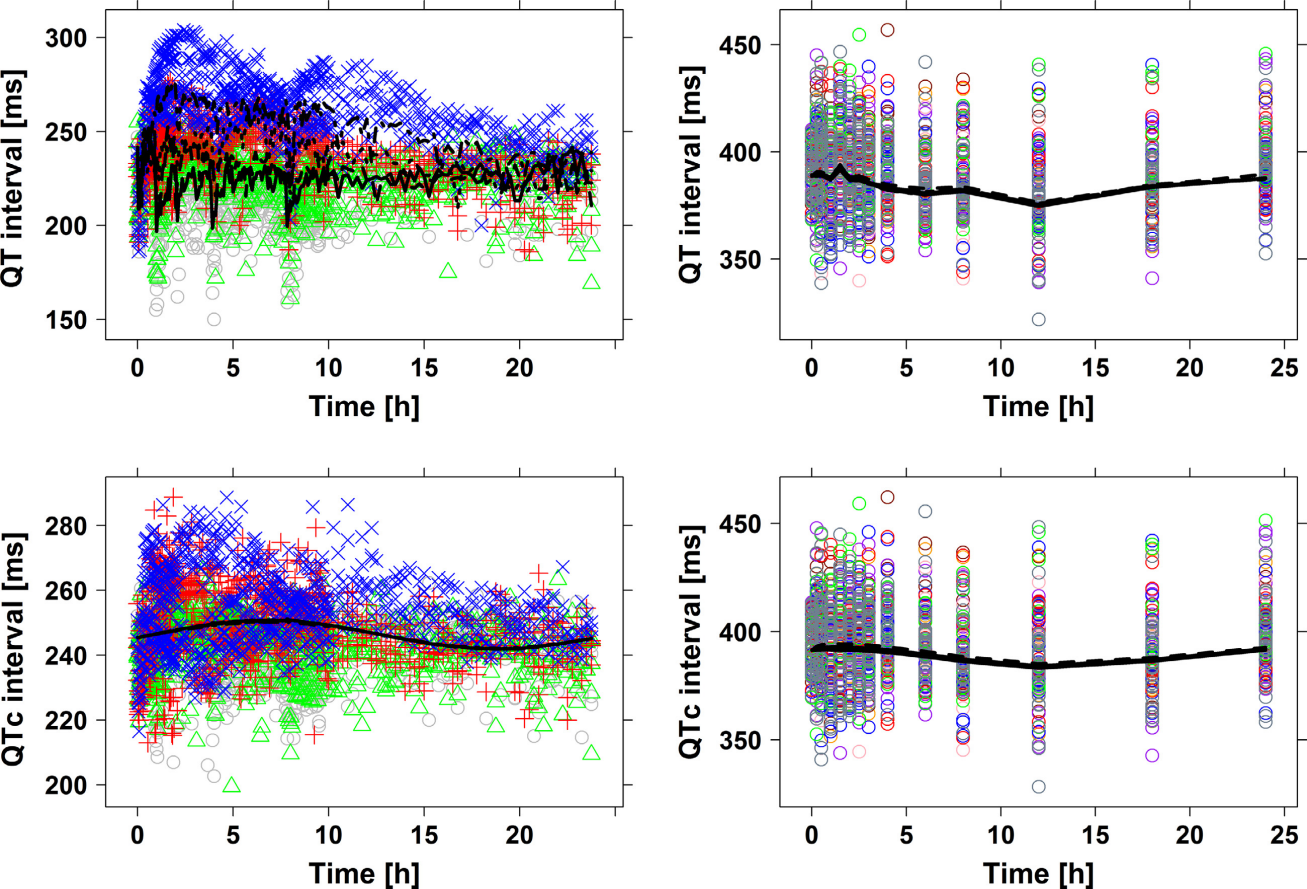
Predicted QT profiles after administration of different doses of methadone to dogs (left) and humans (right). In humans, drug‐induced effects are based on the extrapolation of the slope parameter observed in dogs. Observations are indicated by symbols and population predictions by lines. Time is the time after dose in hours. In dogs ○ (gray) and ^_____^ are predose values; ▵ (red) and ^_ _ _ _^ represent 0.2 mg/kg; + (blue) and ‐ ‐ ‐ ‐ 0.6 mg/kg, ♢ (light blue) and ^__ __ __^ 2 mg/kg methadone. In humans, different colours are used to depict each dose level, namely 5 (green), 10 (red), 25 (blue), 50 (pink), 100 (brown), 250 (purple), and 500 mg (orange) methadone.

The data analysis was performed using the recorded ECG measurements and predicted drug concentrations at the corresponding sampling time. Model parameters are summarized in Table S2 (supplemental results). It is worth mentioning that system‐specific parameters, that is, baseline QT (QT_0_), the QT‐RR correction factor (*α*), the amplitude (A), and phase (Ф) showed values within the same range observed in previous experiments. On the other hand, parameter estimates for *α*, A, and Ф were characterized by relatively low precision. Such a low precision may be caused by a possible delay in the QT adaptation to changes in RR, as shown in Figure 2. Based on the final slope estimates, the model predicted a small but positive QT‐prolonging effect in dogs. This corresponded to a probability of 0 for a QT interval prolongation ≥ 5 msec and ≥10 msec at *C*_max_.

### Simulation scenarios and prediction of the dromotropic effect in humans

Despite the limited experimental data available for the estimation and extrapolation of the slope parameter from dogs to humans, the different scenarios proposed here attempt to account for the uncertainty or lack of precision in this drug‐specific parameter. The slope parameter in humans was therefore derived from the mean and upper 95 credible interval of the estimates obtained in dogs, namely, 0.000019 and 0.059 ms/*μ*M; these values were obtained using the 11.6 translational factor, which describes the differences in effect between the species for (Dubois et al. 2016a,b). Based on these values, QT intervals were simulated using the predicted methadone concentrations at the proposed dose levels in the context of a Phase I dose escalation (FTIH) protocol and a TQT study, as summarized in Figure S1 and in Table S1 (supplemental results).

**Figure 3:**
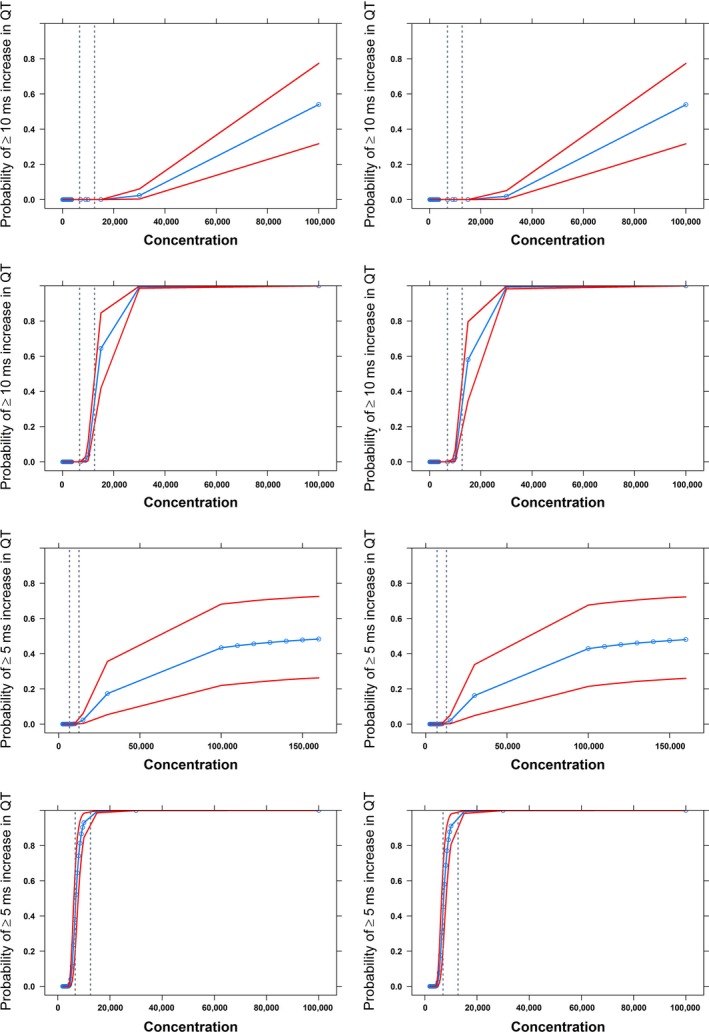
Predicted probability of QT interval prolongation of ≥ 10 msec (upper panels) and ≥5 msec (lower panels) in humans based on a typical FTIH study design. Scenarios include the average (first and third row) and worst case scenario (second and fourth row) for the slope parameter values derived by the extrapolation from dogs. Given the gender differences in QT interval, predictions are stratified by gender: males are shown in the right panels, whereas females are depicted in the left panels. Blue line indicates the mean probability estimate; redlines: 90% credible intervals. The dashed lines indicate the observed *C*_max_ range at the highest dose level (500 mg) used in the simulated study. X‐axis shows concentration in nM.

**Figure 4:**
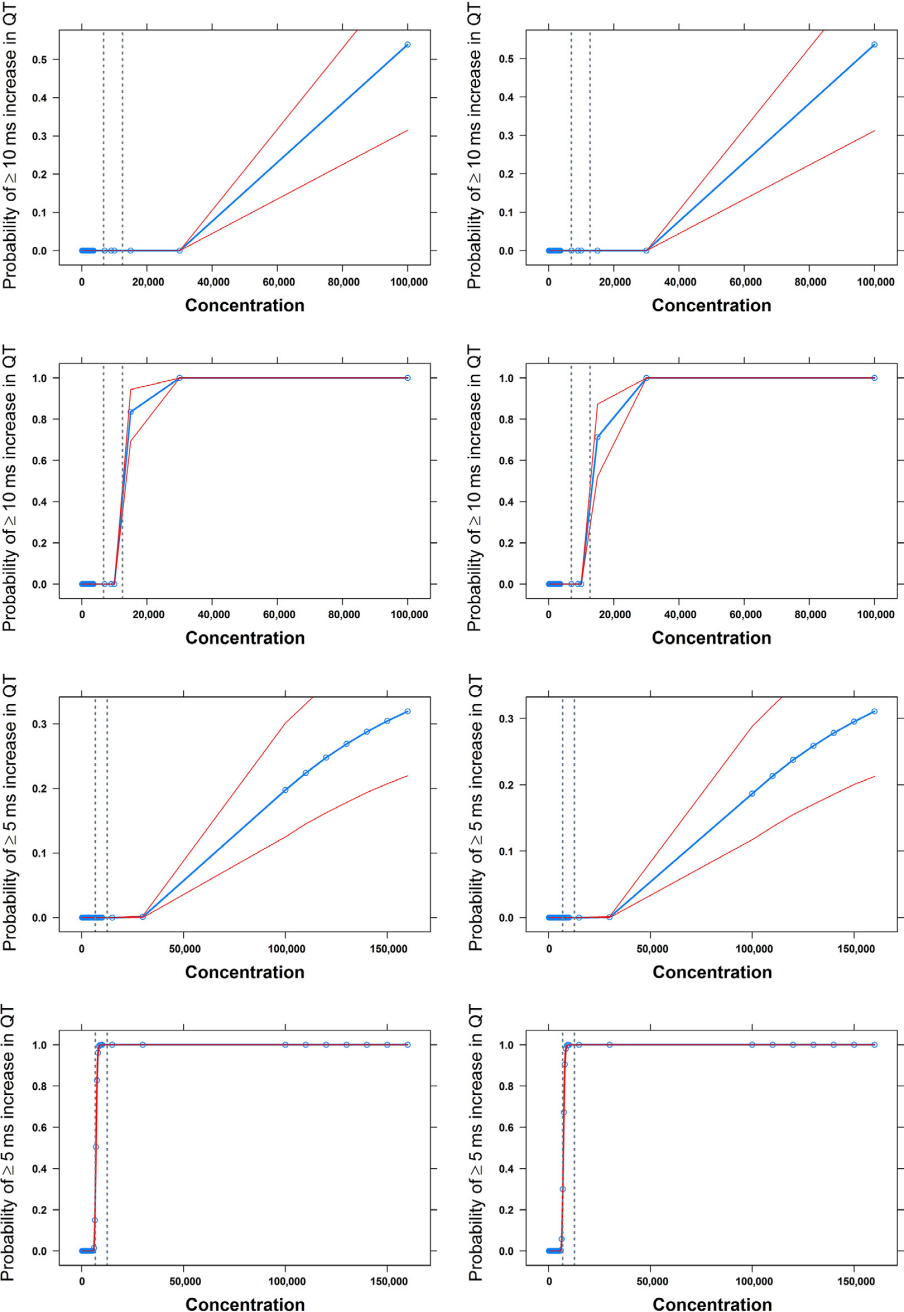
Predicted probability of QT interval prolongation of ≥10 msec (upper panels) and ≥5 msec (lower panels) in humans based on a time‐matched baseline TQT study design. Scenarios include the average (first and third row) and worst case scenario (second and fourth row) for the slope parameter values derived by the extrapolation from dogs. Given the gender differences in QT interval, predictions are stratified by gender: males are shown in the right panels, whereas females are depicted in the left panels. Blue line indicates the mean probability estimate; redlines: 90% credible intervals. The dashed lines indicate the observed *C*_max_ range at the highest dose level (500 mg) used in the simulated study. X‐axis shows concentration in nM.

The results from the simulations are summarized in Figures 3, 4. It should be noted that consistent results with acceptable variability were obtained for system‐specific parameters in all the scenarios (Table 3). The probability curves show that a FTIH is less likely to pick up a possible QT prolongation ≥10 ms in a limited number of subjects, as compared to results observed in a dedicated protocol, in which a fixed supratherapeutic dose level is used across all subjects. On the other hand, the use of different thresholds for QT prolongation has revealed some important features of the dromotropic effects of methadone. Clearly, a different pattern arises from estimates based on a lower threshold for prolongation of 5 ms, as compared to 10 ms. At the predicted peak concentrations, the probability of QT interval prolongation ≥ 5 ms ranged between 0 and 0.51 based on the mean and upper boundary of the 95% credible interval for the slope parameter, respectively. These findings suggest that at therapeutic levels methadone is likely to prolong QT interval, but absolute changes may range between 5 and 10 ms. An overview of these findings is summarized in Table S3 (supplemental results).

## Discussion

The lack of quantitative measures describing the QT‐prolonging effects in preclinical assays can lead to potentially life‐saving drugs being discarded prior to their progression into clinical development, whereas other compounds may reach clinical trials despite their proarrhythmic activity (Wallis 2010). In this investigation we have shown the implementation of a model‐based approach for prospective evaluation of the dromotropic effects of new candidate molecules. Methadone was selected as a paradigm compound, given the long‐standing debate about the magnitude of its QT‐prolonging effects and absence of data regarding hERG inhibition at the time of its development. Using extrapolated estimates from the slope parameter in dogs, we have shown how quantitative pharmacology methods can be used as a tool for experimental protocol optimization and prediction of drug effects at clinically relevant concentrations in humans. Our results also highlight some important points that need to be considered when translating preclinical findings from dogs to humans.

First, it is worth mentioning that drug‐induced QT prolongation is very small in dogs, as expressed by shallow slope and wide credible intervals for the slope parameter. Whereas we have shown in previous investigations that dogs may be less sensitive to QT‐prolonging effects (Chain and Dubois et al. 2013; Dubois et al. 2014, 2016a,b), we believe that the lower exposure levels achieved in dogs also played an important role in the precision of the parameter estimates.

It is well established that not only the physiological (baseline) QT values are very different between dogs and humans, but also that sensitivity to the drug effect on the QT interval varies between the two species. This is reflected in the slope of the linear drug–concentration–effect relationship, where the slope of the curve reflects the sensitivity to the drug in terms of the change in QT interval (in ms) per unit of drug concentration (e.g., in nM). In a previous investigation, analyzing the effects of 10 different drugs, it has been demonstrated that there is a mean 11.6‐fold difference in the slope of the concentration–effect relation between dogs and humans (Dubois et al. 2016a,b). In this investigation this same factor was used to predict the effect of methadone on the QT in humans from the slope parameter estimate in dogs.

The 95% CI for QTc0 in this study was considerably large. We have no clear explanation for the observed variability. An important observation in this respect is that correction for heart rate does not result in an appreciable reduction in the variability, as shown in Figure 2, where the time courses of both QT and QTc are presented in the lower left panel. In this study an individual correction factor for RR was calculated by an individual exponent (*α*) per subject/animal. The use of alternative methods for the heart rate correction (i.e., fixed exponents as Fridericia or Bazett) did not result in further reduction in the variation. Despite the variation in the data, the model was able to describe the concentration–effect relationship with sufficient precision to obtain realistic parameter estimates. In addition, given that our protocols were performed in a blinded manner, experiments in dogs were designed according to a typical safety pharmacology protocol, without correcting for the currently known interspecies differences in drug metabolism. Such a mismatch occurs despite current recommendations by ICH S7A, which aim at plasma exposure levels that ‘include and exceed the primary pharmacodynamic or therapeutic range’ (International committee of Harmonisation 2000).

### Interspecies differences in drug disposition

Differences or discrepancies in pharmacokinetic properties are not a unique feature of methadone (Garrett et al. 1985). Therefore, attention to the dose rationale in experimental protocols is crucial for accurate extrapolation, translation, and interpretation of proarrhythmic or dromotropic effects in preclinical species. On the other hand, the striking differences in the pharmacokinetics of methadone between dogs and humans make this case a very interesting point for discussion from a drug development perspective (Florian et al. 2012). We have thus far endorsed the views that characterization of PKPD relationships in animals in conjunction with early PKPD data in humans should provide sufficient evidence about the probability of QT interval prolongation ≥10 ms in humans, without relying on the need for a TQT study as the final confirmatory step. Our findings seem to support that view and raise a major concern about the role of clinical pharmacology in safety evaluation. Despite having a consolidated role in regulatory processes, the use of quantitative pharmacology concepts in preclinical safety research remains very limited. Of particular relevance is the possibility of using PKPD relationships in conjunction with allometric scaling principles to support dose selection in toxicology and safety pharmacology experiments (Sahota et al. 2014).

Another interesting feature of this exercise with methadone are the differences in enantioselective metabolism observed between species, which applies to a range of compounds with chiralic properties or which yield metabolites with affinity for hERG or other ion channels. According to Eap et al. (2007) the (S)‐enantiomer of methadone has a significantly higher affinity for hERG compared with (R)‐methadone (see Fig. 2S in supplemental results). Interestingly, in humans CYP2B6 has been found to be a major contributor to the elimination of methadone, which primarily metabolizes (S)‐methadone (Chang et al 2011). By contrast, in dogs this CYP2B isozyme is not available (Martignoni et al. 2006). In theory, the presence of higher levels of (S)‐methadone in humans as compared to dogs, lower clearance, and a difference in potency of the two moieties (2 vs. 7 *μ*M for (S)‐ and (R)‐methadone) would be sufficient to explain differences in the QT prolonging effects between dogs and humans (Florian et al. 2012). Unfortunately, in the current drug screening paradigm barely any of the aforementioned points are accounted for in a quantitative manner when assessing the proarrhythmic risk of a candidate molecule (Brocks 2006; Hutt 2007). In these circumstances, the use of a model‐based approach will not replace hard evidence of experimental data, but can be used as a tool to explore the impact of interspecies differences. Inferential methods are available that would allow assessment of a range of simulation scenarios based on previously observed class effects or by simple sensitivity analysis.

### Drug‐specific parameters as the basis for translating preclinical findings

The methadone example offers another important insight into the requirements for translational research. The predictive performance of a PKPD model or the predictive value of experimental findings in humans cannot be taken for granted without further understanding of the processes underpinning drug disposition and mechanism of action, that is, the underlying substrates. We have demonstrated that a correlation can be established between drug‐specific parameters in dogs and humans. In fact, the rationale for a correlation between the slope parameter is supported by evidence showing that the canine Ether‐a‐Go‐Go (cERG) potassium channel plays the same role in the action potential repolarization, contributing to the drug‐related QT prolongation with selectivity and sensitivity somewhat comparable to the channel in humans hERG (Haushalter et al. 2008). Such a correlation has been defined under the assumption that hERG inhibition by the parent compound explains the observed changes in QT interval in both species. Consequently, after correcting for differences in drug disposition, any discrepancies in the magnitude of the proarrhythmic effect of a compound in dogs and humans may be assigned to intrinsic differences in the biological systems, for example, hERG and ion channel density.

Our simulation scenarios do not predict the same effect size described by Florian et al. (2012). In their work, a 10‐ms increase was associated with *C*_max_ values observed after administration of a 200‐mg dose of methadone. By contrast, at this dose level the predicted effect in healthy subjects was marginal; our results suggest a 50% probability of ≥5 msec increase around the *C*_max_ after a 500 mg dose. Such a discrepancy does not necessarily represent inaccuracies in the estimates of the drug‐specific properties, but seem to reflect the differences in methadone disposition in humans (i.e., enantioselective metabolism) as well as other clinical factors. In fact, we have previously shown that considerable differences may exist between drug‐induced effects in controlled clinical trials and observed QT prolongation in real‐life conditions (Chain et al. 2013). In addition, one should recognize that the analysis of PKPD data based on a limited range of doses may lead to overestimation of drug‐specific parameters (e.g., slope). This limitation can be seen in the work by Roy et al. (2011), who have shown drug‐induced‐corrected QT interval prolongation in patients receiving relatively low daily doses of methadone therapy, with no evidence of a dose–response relationship. Even though an exercise involving the translation from animal to humans to real‐life conditions was out of the scope of our work, the use of a worst case scenario for slope estimates allowed us to describe effect sizes in line with those reported by Florian et al. (2012) and Martell et al. (2005). The observed effect was detectable only in the context of a TQT study most likely due to the inclusion of both therapeutic and supratherapeutic exposures of methadone. A summary of the limitations of the current approach and perspectives for improved cardiovascular safety can be founded in the supplemental material S1.

## Conclusions

The objective of our study was to demonstrate that QT interval prolongation in humans can be predicted from preclinical data in conscious dogs, by extrapolating the slope of the linear concentration–effect relationship, which differs from humans by a factor of 11.6 (Dubois et al. 2016a,b). In addition, our approach enables the characterization of different thresholds, as shown for prediction of a probability of a 10‐ms and 5‐ms prolongation.

The results from this extensive set of simulations also support the use of preclinical data from safety pharmacology protocols in dogs to detect the prolongation of QT interval in humans irrespective of the drug effect size. Our findings illustrate how concentration–QT prolongation relationships can be extrapolated from dogs and combined with data from FTIH studies, enabling the characterization of the safety profile of a compound in early clinical development. This approach is in alignment with the activities suggested by the CSCR/IQ consortium, which is aimed at establishing the feasibility of incorporating QT measurements to single and multiple ascending doses (SAD/MAD) studies and to replace TQT studies (Darpo et al. 2014). On the other hand, this exercise made clear that differences in drug disposition as well as selectivity of action needs to be considered for the accurate translation of experimental findings from preclinical species to humans. These factors need to be accounted for, but are often overlooked during the progression of candidate molecules into clinical development.

We recommend the adoption of the approach by R&D as it may provide the opportunity to further validate the correlation between drug‐specific parameters in dogs and humans. In addition, it has become evident that availability of relevant data on drug disposition is essential for the accurate estimation of the probability of QT prolongation in humans.

## Disclosure

There are no conflicts of interest.

## Supporting information

Supplemental Results
**Table S1.** Pharmacokinetic parameter estimates used for the simulation of methadone concentrations in dogs and healthy subjects.
**Table S2.** Mean PKPD parameter estimates and 95% credible intervals obtained after oral administration of methadone to dogs (*n* = 4).
**Table S3.** Mean PKPD parameter estimates and 95% credible intervals obtained from the simulation of the dromotropic effects of methadone in FTIH and TQT studies, including scenarios in which baseline and time‐matched baseline analysis are presented.
**Figure S1.** Upper panels show individual RR profiles over time and the potential impact of direct drug levels on heart rate. Lower panel depicts the individual PKPD relationships (QT interval vs. predicted methadone concentration) in dogs (left) and humans (right). Time is the time after dose in hours. In dogs (left), doses of 0.2, 0.6, and 2 mg/kg methadone are depicted in green, red, and blue, respectively. In humans (right), simulated data mimic a cohort of 27 subjects. Doses of 5, 10, 25, 50, 100, 250 and 500 mg methadone are depicted in green, red, blue, pink, brown, purple, and orange, respectively.
**Figure S2.** hERG inhibition curves for racemic, (R)‐ and (S)‐methadone. Reprinted with permission from Eap et al (2007).

**Supplemental Material.** Summary of the bioanalytical method, pharmacokinetic (PK) and pharmacokinetic‐pharmacodynamic (PKPD) modeling and extrapolation procedures, including a discussion of the limitations and perspectives for the use of a model‐based approach for the assessment of QT interval prolongation in early drug development.

## References

[prp2284-bib-1000] Bellanti F , Chain A , Danhof M , Della Pasqua O . Relevance of QT‐RR correlations in the assessment of QTc‐interval prolongation in clinical trial simulations, PAGE 20 (2011) (http://www.page-meeting.org/?abstract=2168; last accessed on 20/10/16).

[prp2284-bib-1001] Brocks DR (2006). Drug disposition in three dimensions: an update on stereoselectivity in pharmacokinetics. Biopharm Drug Dispos. 27: 387–406. 16944450 10.1002/bdd.517

[prp2284-bib-0001] Cavero I , Holzgrefe H (2015). CiPA: ongoing testing, future qualification procedures, and pending issues. J Pharmacol Toxicol Methods 76: 27–37. 26159293 10.1016/j.vascn.2015.06.004

[prp2284-bib-1002] Chain AS , Krudys KM , Danhof M , Della Pasqua O (2011). Assessing the probability of drug‐induced QTc‐interval prolongation during clinical drug development. Clin Pharmacol Ther 90: 867–75. 22048226 10.1038/clpt.2011.202

[prp2284-bib-0003] Chain ASY , Dubois VFS , Danhof M , Sturkenboom MCJ , Della Pasqua O (2013). Identifying the translational gap in the evaluation of drug‐induced QTc‐interval prolongation. Br J Clin Pharmacol 76: 708–724. 23351036 10.1111/bcp.12082 PMC3853530

[prp2284-bib-0004] Chain ASY , Dieleman JP , Van Noord C , Hofman A , Stricker BHC , Danhof M , et al. (2013). Not‐in‐trial simulation I : bridging cardiovascular risk from clinical trials to real‐life conditions. Br J Clin Pharmacol 76: 964–972. 23617533 10.1111/bcp.12151 PMC3845320

[prp2284-bib-1003] Chang Y , Fang WB , Lin S‐N , Moody DE (2011). Stereo‐selective metabolism of methadone by human liver microsomes and cDNA‐expressed cytochrome P450s: A Reconciliation. Basic Clin Pharmacol Toxicol. 108: 55–62. 20825389 10.1111/j.1742-7843.2010.00628.x PMC3005981

[prp2284-bib-0005] Codd EE , Shank RP , Schupsky JJ , Raffa RB (1995). Serotonin and norepinephrine uptake inhibiting activity of centrally acting analgesics: structural determinants and role in antinociception. J Pharmacol Exp Ther 274: 1263–1270. 7562497

[prp2284-bib-0006] Dale OLA , Hoffer C , Sheffels P , Kharasch ED (2002). Disposition of nasal, intravenous, and oral methadone in healthy volunteers. Clin Pharmacol Ther 72: 536–545. 12426517 10.1067/mcp.2002.128386

[prp2284-bib-0007] Dale OLA , Sheffels P , Kharasch ED (2004). Bioavailabilities of rectal and oral methadone in healthy subjects. Br J Clin Pharmacol 58: 156–162. 15255797 10.1111/j.1365-2125.2004.02116.x PMC1884589

[prp2284-bib-0008] Darpo B , Garnett C , Benson CT , Keirns J , Leishman DJ , Marek M , et al. (2014). Cardiac safety research consortium: can the thorough QT/QTc study be replaced by early QT assessment in routine clinical pharmacology studies? Scientific update and a research proposal for a path forward. Am Heart J 168: 262–272. 25173536 10.1016/j.ahj.2014.06.003

[prp2284-bib-0009] Davies B , Morris T (1993). Physiological parameters in laboratory animals and humans. Pharm Res 10: 1093–1095. 8378254 10.1023/a:1018943613122

[prp2284-bib-0010] Dubois VFS , Yu H , Danhof M , Della Pasqua O (2014). Model‐based evaluation of drug‐induced QT(c) prolongation for compounds in early development. Br J Clin Pharmacol 79: 148–161. 10.1111/bcp.12482 PMC4294084 25099645

[prp2284-bib-0011] Dubois VFS , de Witte WEA , Visser SAG , Danhof M , Della Pasqua O (2016a). Assessment of interspecies differences in drug‐induced QTc interval prolongation in cynomolgus monkeys, dogs and humans. Pharm Res 33: 40–51. 26553352 10.1007/s11095-015-1760-9 PMC4689776

[prp2284-bib-0012] Dubois VFS , Smania G , Yu H , Graf R , Chain ASY , Danhof M , et al. (2016b). Translating QT interval prolongation from conscious dogs to humans. Br J Clin Pharmacol. doi:10.1111/bcp.13123. 10.1111/bcp.13123 PMC5237692 27614058

[prp2284-bib-0013] Eap CB , Crettol S , Rougier J‐S , Schläpfer J , Sintra Grilo L , Déglon J‐J , et al. (2007). Stereoselective block of hERG channel by (S)‐methadone and QT interval prolongation in CYP2B6 slow metabolizers. Clin Pharmacol Ther 81: 719–728. 17329992 10.1038/sj.clpt.6100120

[prp2284-bib-0014] Food and Drug Administration . 2005 “Guidance for Industry: E14 Clinical Evaluation of QT/QTc Interval Prolongation and Proarrhythmic Potential for Non‐Antiarrhythmic Drugs.” US Department of Health and Human Services. (http://www.fda.gov/ohrms/dockets/ac/06/briefing/2006-4254b_08_06_qt%20guidance.pdf ‐ last accessed on 10/10/16).

[prp2284-bib-0015] Fermini B , Hancox JC , Abi‐Gerges N , Bridgland‐Taylor M , Chaudhary KW , Colatsky T , et al. (2016). A new perspective in the field of cardiac safety testing through the comprehensive in vitro proarrhythmia assay paradigm. J Biomol Screen 21: 1–11. 26170255 10.1177/1087057115594589

[prp2284-bib-0016] Florian J , Garnett CE , Nallani SC , Rappaport BA , Throckmorton DC (2012). A modeling and simulation approach to characterize methadone QT prolongation using pooled data from five clinical trials in MMT patients. Clin Pharmacol Ther 91: 666–672. 22378153 10.1038/clpt.2011.273

[prp2284-bib-0019] France NP , Della Pasqua O (2015). The role of concentration−effect relationships in the assessment of QTc interval prolongation. Br J Clin Pharmacol 79: 117–131. 24938719 10.1111/bcp.12443 PMC4294082

[prp2284-bib-0021] Garrett ER , Derendorf H , Mattha AG (1985). Pharmacokinetics of morphine and its surrogates VII: high‐performance liquid chromatographic analyses and pharmacokinetics of methadone and its derived metabolites in dogs. J Pharm Sci 74: 1203–1214. 4087182 10.1002/jps.2600741114

[prp2284-bib-0022] Gorman AL , Elliott KJ , Inturrisi CE (1997). The d‐ and l‐isomers of methadone bind to the non‐competitive site on the N‐methyl‐D‐aspartate (NMDA) receptor in rat forebrain and spinal cord. Neurosci Lett 223: 5–8. 9058409 10.1016/s0304-3940(97)13391-2

[prp2284-bib-0024] Haushalter TM , Friedrichs GS , Reynolds DL , Barecki‐Roach M , Pastino G , Hayes R , et al. (2008). The cardiovascular and pharmacokinetic profile of dofetilide in conscious telemetered beagle dogs and cynomolgus monkeys. Br J Pharmacol 154: 1457–1464. 18604237 10.1038/bjp.2008.275 PMC2492096

[prp2284-bib-1004] Hutt AJ (2007). Chirality and pharmacokinetics: an area of neglected dimensionality? Drug Metabol Drug Interact. 22: 79–112. 17708062 10.1515/dmdi.2007.22.2-3.79

[prp2284-bib-0025] International Conference on Harmonisation . 2000 “ICH S7A. Saftey Pharmacology Studies for Human Pharmaceuticals” In International Conference on Harmonisation of Technical Requirements For Registration of Pharmaceuticals For Human Use. (http://www.ich.org/fileadmin/Public_Web_Site/ICH_Products/Guidelines/Safety/S7A/Step4/S7A_Guideline.pdf ‐ last accessed on 10/10/16)

[prp2284-bib-0026] Kukanich SP (2005). Pharmacokinetics and its application to dosage design in veterinary medicine. PhD Thesis, North Carolina State University, North Carolina, USA (http://www.lib.ncsu.edu/resolver/1840.16/3509 ‐ last accessed on 10/10/16).

[prp2284-bib-0027] Kukanich B , Borum SL (2008). The disposition and behavioral effects of methadone in greyhounds. Vet Anaesth Analg 35: 242–248. 18282261 10.1111/j.1467-2995.2007.00369.x

[prp2284-bib-0028] Martell BA , Amsten JH , Krantz MJ , Gourevitch MN (2005). Impact of methadone treatment on cardiac repolarization and conduction in opioid users. Am J Cardiol 95: 915–918. 15781034 10.1016/j.amjcard.2004.11.055

[prp2284-bib-0029] Martignoni M , Groothuis GMM , De Kanter R (2006). Species differences between mouse, rat, dog, monkey and human CYP‐mediated drug metabolism, inhibition and induction. Expert Opin Drug Metab Toxicol 2: 875–894. 17125407 10.1517/17425255.2.6.875

[prp2284-bib-0030] Meresaar U , Nilsson M , Holmstrand J , Anggard E (1981). Single dose pharmacokinetics and bioavailability of methadone in man studied with a stable isotope method. Eur J Clin Pharmacol 20: 473–478. 7286059 10.1007/BF00542102

[prp2284-bib-0031] Mujtaba S , Romero J , Taub CC . (2013). Methadone, QTc prolongation and torsades de pointes: current concepts, management and a hidden twist in the tale? J Cardiovasc Dis Res 4 Elsevier Ltd: 229–235. 24653586 10.1016/j.jcdr.2013.10.001 PMC3953689

[prp2284-bib-0032] Payte JT (1991). A brief history of methadone in the treatment of opioid dpendence : a personal perspective. J Psychoactive Drugs 23: 103–107. 1765883 10.1080/02791072.1991.10472226

[prp2284-bib-0034] Roy AK , McCarthy C , Kiernan G , McGorrian C , Keenan E , Mahon NG , et al. (2011). Increased incidence of QT interval prolongation in a population receiving lower doses of methadone maintenance therapy. Addiction 107: 1132–1139. 10.1111/j.1360-0443.2011.03767.x 22168435

[prp2284-bib-0035] Sager PT , Gary Gintant J , Turner R , Pettit S , Stockbridge N (2014). Rechanneling the cardiac proarrhythmia safety paradigm: a meeting report from the cardiac safety research consortium. Am Heart J 167 Mosby, Inc.: 292–300. 24576511 10.1016/j.ahj.2013.11.004

[prp2284-bib-0036] Sahota T , Sanderson I , Danhof M , Della Pasqua OE (2014). Model‐based analysis of thromboxane B2 and prostaglandin E2 as biomarkers in the safety evaluation of naproxen. Toxicol Appl Pharmacol 278: 209–219. 24667227 10.1016/j.taap.2014.03.010

[prp2284-bib-0037] Selley DE , Cao CC , Sexton T , Schwegel JA , Martin TJ , Childers SR (2001). *μ*‐opioid receptor‐mediated G‐protein activation by heroin metabolites: evidence for greater efficacy of 6‐monoacetylmorphine compared with morphine. Biochem Pharmacol 62: 447–455. 11448454 10.1016/s0006-2952(01)00689-x

[prp2284-bib-0038] Shah RR (2007). Cardiac repolarisation and drug regulation: assessing cardiac safety 10 years after the CPMP guidance. Drug Saf 30: 1093–1110. 18035863 10.2165/00002018-200730120-00003

[prp2284-bib-0039] Totah RA , Sheffels P , Roberts T , Whittington D , Thummel K , Kharasch ED (2008). Role of CYP2B6 in stereoselective human methadone metabolism. Anesthesiology 108: 363–374. 18292673 10.1097/ALN.0b013e3181642938

[prp2284-bib-0040] Wallis RM (2010). Integrated risk assessment and predictive value to humans of non clinical repolarization assays. Br J Pharmacol 159: 115–121. 19785646 10.1111/j.1476-5381.2009.00395.x PMC2823357

[prp2284-bib-0041] Wang J‐S , De Vane Lindsay C (2003). Involvement of CYP3A4, CYP2C8, and CYP2D6 in the metabolism of (R)‐ and (S)‐methadone in vitro. Drug Metab Dispos 31: 742–747. 12756206 10.1124/dmd.31.6.742

