# Supplementary material for: Characterizing QT interval prolongation in early clinical development: a case study with methadone
Source: Pharmacol Res Perspect. 2017 Jan 24;5(1):e00284. doi: 10.1002/prp2.284 (PMC5461648; doi:10.1002/prp2.284)
Supplement: Supplementary file 1 — Supplemental Results Table S1. Pharmacokinetic parameter estimates used for the simulation of methadone concentrations in dogs and healthy subjects. Table S2. Mean PKPD parameter estimates and 95% credible intervals obtained after oral administration of methadone to dogs (n = 4). Table S3. Mean PKPD parameter estimates and 95% credible intervals obtained from the simulation of the dromotropic effects of methadone in FTIH and TQT studies, including scenarios in which baseline and time‐matched baseline analysis are presented. Figure S1. Upper panels show individual RR profiles over time and the potential impact of direct drug levels on heart rate. Lower panel depicts the individual PKPD relationships (QT interval vs. predicted methadone concentration) in dogs (left) and humans (right). Time is the time after dose in hours. In dogs (left), doses of 0.2, 0.6, and 2 mg/kg methadone are depicted in green, red, and blue, respectively. In humans (right), simulated data mimic a cohort of 27 subjects. Doses of 5, 10, 25, 50, 100, 250 and 500 mg methadone are depicted in green, red, blue, pink, brown, purple, and orange, respectively. Figure S2. hERG inhibition curves for racemic, (R)‐ and (S)‐methadone. Reprinted with permission from Eap et al (2007). [file PRP2-5-e00284-s001.pdf]

Supplemental results

Table S1: Pharmacokinetic parameter estimates used for the simulation of (racemic) methadone concentrations in dogs and healthy subjects.

	Dogs		Healthy subjects	
**Parameters**	Mean	IIV(%)	Mean	IIV(%)
**Ka (h^{-1})**	11.6		0.036	
**CL (L/h)**	41.1	38.5	13	
**V (L)**	247	30.8	4	54.8
**Error**	0.00011			

Table S2: Mean PKPD parameter estimates and 95% credible intervals obtained after oral administration of methadone to dogs (n=4).

**Model parameters**	
**QTc_0 [ms]**	246 (120 - 490)
**A**	0.23 (0.12 - 0.48)
**A [ms]**	4.04 (1.7 - 9.3)
**ϕ [h]**	15.77 (7.02 - 34.64)
**Slope [ms/uM]**	1.9E-05 (-0.062 - 0.059)
**BSV (QTc_0) %**	59 (30 - 264)
**BSV (α) %**	59 (31 - 280)
**BSV (A) %**	73(37 - 412)
**BSV (ϕ) %**	69 (35 - 373)
**BSV (Slope) %**	0.00075 (0.00037 – 0.0023)
**Residual Error [ms]**	9.5 (9.3 – 9.8)
**Prob Effect $\geq$ 10 ms at Cmax**	0
**Cmax [nM]**	474

Table S3: Mean PKPD parameter estimates and 95% credible intervals obtained from the simulation of the dromotropic effects of methadone in FTIH and TQT studies, including scenarios in which baseline and time-matched baseline analysis are presented. Predicted drug concentrations in humans and RR data were used in conjunction with extrapolations of the slope parameter observed in dogs. The interspecies correlation for this drug-specific parameter is based on the assumption that QT prolongation is determined by hERG inhibition mechanisms. Average= scenarios in which mean PKPD parameter estimates are used; WTC= worst case scenario, in which the upper limit of the 95% credible intervals are used.

Parameter	FTIH average 10ms	FTIH average 5ms	FTIH WCS 10ms	FTIH WCS 5ms
**Slopem (ms/µM)**	0.061 (-0.22-0.32)	0.063 (-0.22-0.319	0.661 (0.4-1.045)	0.661 (0.4-1.045)
**Slopef (ms/µM)**	6.37E-05 (-0.23-332)	0.0655 (-0.23-0.332)	0.686 (0.415-1.086)	0.686 (0.414-1.086)
**Alpha (α)**	0.338 (0.318-0.360)	0.338 (0.318-0.360)	0.338 (0.317-0.360)	0.338 (0.317-0.360)
**Amplitude (ms)**	4.20 (3.64-4.85)	4.20 (3.64-4.85)	4.22 (3.65-4.87)	4.22 (3.65-4.87)
**Phase (h)**	9.93 (9.38-10.52)	9.93 (9.38-10.52)	9.92 (9.38-10.51)	9.92 (9.38-10.51)
**QTc0m (ms)**	387.3 (357.9-419.0)	387.3 (357.9-419.0)	387.3 (357.8-419.1)	387.3 (357.8-419.1)
**QTc0f (ms)**	402.3 (371.6-435.4)	402.3 (371.6-435.4)	402.3 (371.6-435.4)	402.3 (371.6-435.4)

Parameter	TQT crossover time-matched BL average 10ms	TQT crossover time-matched BL average 5ms	TQT crossover time-matched BL WCS 10ms	TQT crossover time-matched BL WCS 5ms
**Slopem (ms/µM)**	0.262 (0.083-0.41)	0.262 (0.083-0.41)	0.67 (0.514-0.888)	0.689 (0.514-0.888)
**Slopef (ms/µM)**	0.272 (0.0865-0.425)	0.272 (0.0866-0.425)	0.696 (0.534-0.921)	0.715 (0.534-0.921)
**Alpha (α)**	0.339 (0.325-0.353)	0.339 (0.325-0.353)	0.338 (0.324-0.352)	0.338 (0.324-0.352)
**Amplitude (ms)**	3.13 (2.81-3.45)	3.13 (2.81-3.45)	3.13 (2.82-3.45)	3.18 (2.82-3.45)
**Phase (h)**	10.38 (9.97-10.83)	10.38 (9.97-10.83)	10.37 (9.96-10.82)	10.35 (9.96-10.82)
**QTc0m (ms)**	387.4 (367.4-408.4)	387.4 (367.4-408.4)	387.4 (367.5-408.2)	387.4 (367.5-408.2)
**QTc0f (ms)**	402.3 (382.1-423.5)	402.3 (382.1-423.5)	402.3 (381.8-423.78)	402.3 (381.8-423.8)

Parameter	TQT crossover pre dose-baseline average 10ms	TQT crossover pre dose-baseline WCS 10ms	TQT parallel time-matched baseline average 10ms	TQT parallel time-matched baseline WCS 10ms
**Slopem (ms/µM)**	0.264 (0.0846-0.411)	0.691 (0.514-0.889)	0.242 (0.0838-0.40)	0.669 (0.518-0.876)
**Slopef (ms/µM)**	0.274 (0.0879-0.426)	0.717 (0.534-0.921)	0.251 (0.0870-0.42)	0.695 (0.539-0.909)
**Alpha (α)**	0.338 (0.324-0.353)	0.338 (0.324-0.353)	0.338 (0.327-0.350)	0.338 (0.327-0.350)
**Amplitude (ms)**	3.12 (2.80-3.45)	3.11 (2.79-3.45)	3.13 (2.87-3.39)	3.13 (2.87-3.39)
**Phase (h)**	10.38 (9.96-10.83)	10.38 (9.86-10.83)	10.38 (10.05-10.74)	10.37 (10.04-10.74)
**QTc0m (ms)**	387.4 (367.6-408.4)	387.43 (367.4-408.6)	387.3 (371.7-403.5)	387.3 (371.6-403.6)
**QTc0f (ms)**	402.4 (381.9-423.8)	402.4 (382.1-423.6)	402.4 (386.2-418.9)	402.4 (386.3-418.9)

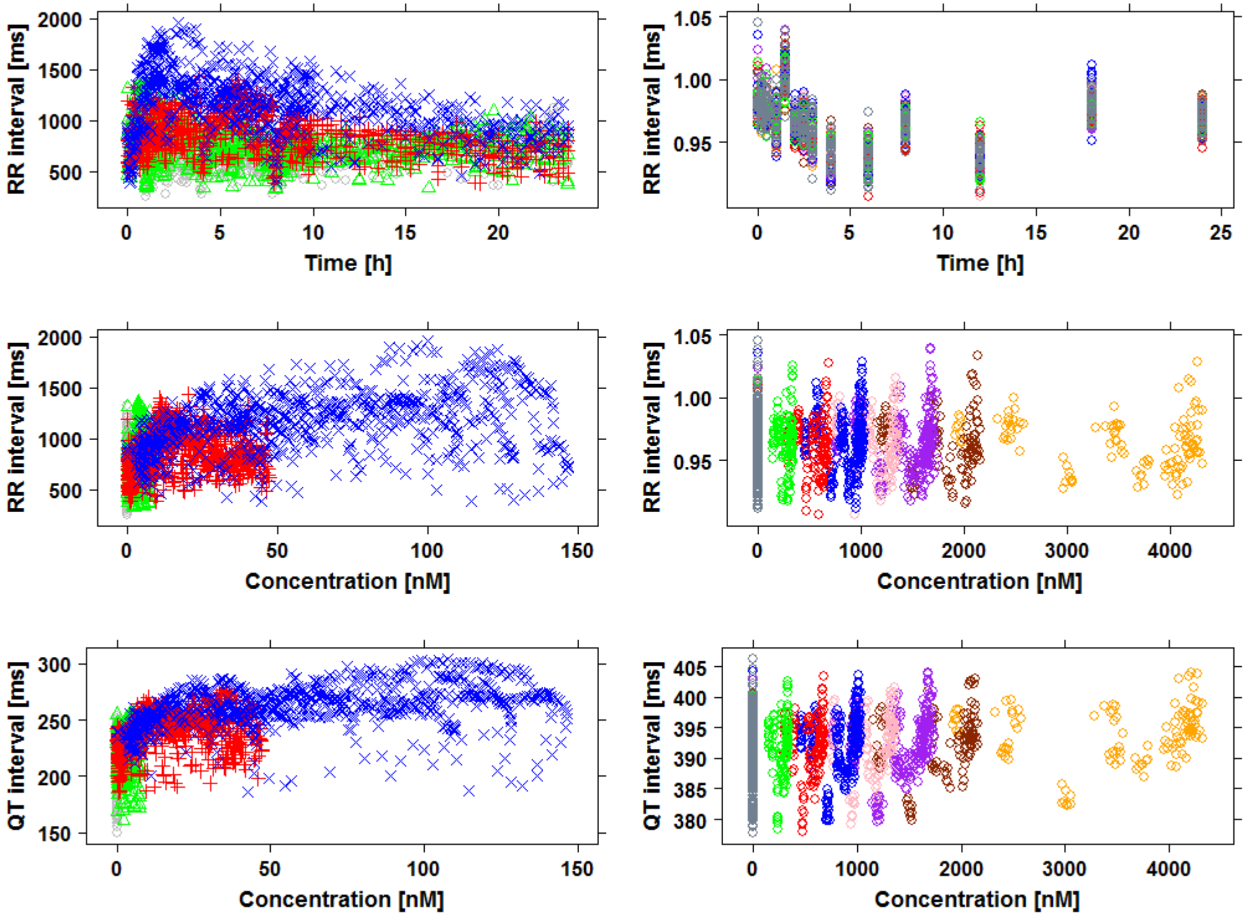

Figure S1: Upper panels show individual RR profiles over time and the potential impact of direct drug levels on heart rate. Lower panel depicts the individual PKPD relationships (QT interval vs. predicted methadone concentration) in dogs (left) and humans (right). Time is the time after dose in hours. In dogs (left), doses of 0.2, 0.6, and 2 mg/kg methadone are depicted in green, red, and blue, respectively. In humans (right), simulated data mimic a cohort of 27 subjects. Doses of 5, 10, 25, 50, 100, 250 and 500 mg methadone are depicted in green, red, blue, pink, brown, purple, and orange, respectively.

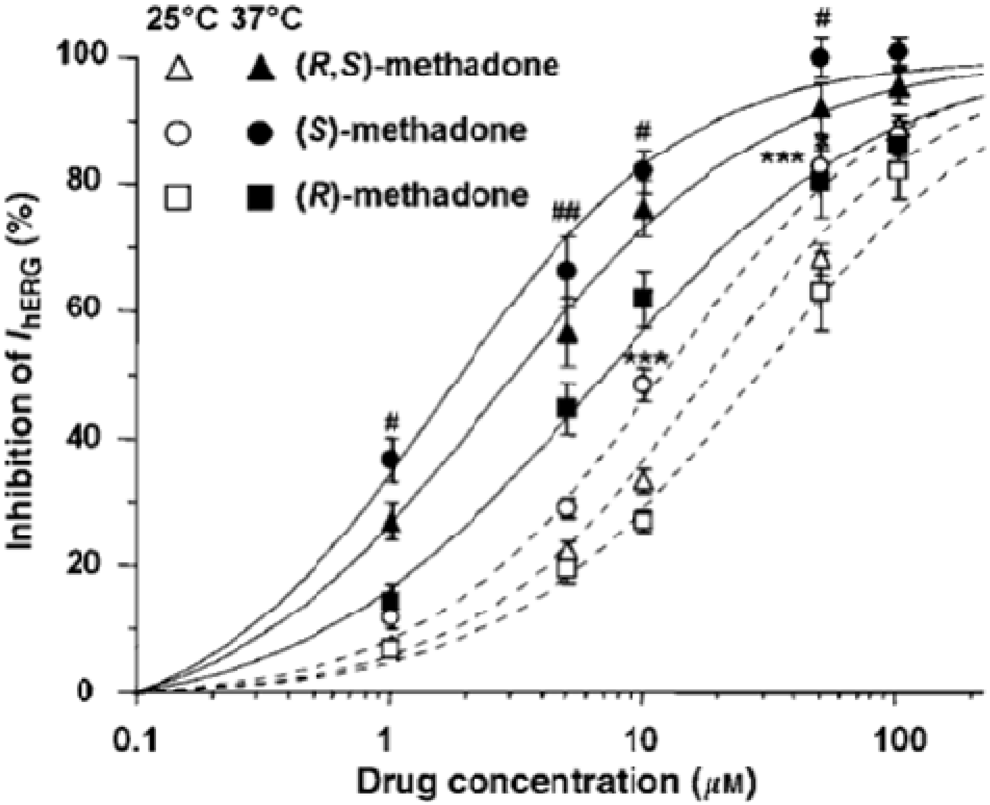

Figure S2: hERG inhibition curves for racemic, (R)- and (S)-methadone. Reprinted with permission from Eap et al (2007)
