# Supplementary material for: Characterizing QT interval prolongation in early clinical development: a case study with methadone
Source: Pharmacol Res Perspect. 2017 Jan 24;5(1):e00284. doi: 10.1002/prp2.284 (PMC5461648; doi:10.1002/prp2.284)
Supplement: Supplementary file 2 — Supplemental Material. Summary of the bioanalytical method, pharmacokinetic (PK) and pharmacokinetic‐pharmacodynamic (PKPD) modeling and extrapolation procedures, including a discussion of the limitations and perspectives for the use of a model‐based approach for the assessment of QT interval prolongation in early drug development. [file PRP2-5-e00284-s002.pdf]

Supplemental material

Methadone Bioanalysis

Blood samples (approximately 1.0 mL per sampling time) taken from a cephalic vein of all animals dosed with vehicle or methadone were collected into tubes containing EDTA mixed gently and then placed on crushed water ice. Five blood sampling times were collected for each animal at 1, 2, 4, 8, and 24 hours after the start of dosing. Two aliquots (50 µL) of each blood sample were accurately dispensed into micronic tubes containing an equal volume of pure water (50 µL) and frozen immediately over solid carbon dioxide. The diluted blood samples were stored at *ca* -80°C until transported.

Blood samples were analyzed for methadone using a validated analytical method based on protein precipitation, followed by HPLC-MS/MS analysis. The lower limit of quantification (LLQ) for Methadone was 1 ng/mL using a 25uL aliquot of dog blood with a higher limit of quantification (HLQ) of 1000. The computer systems that were used on this study to acquire and quantify data included Analyst Version 1.4.2 and SMS2000 version 2.0.

Pharmacokinetic (PK) modelling

As time-matched concentration and QT interval values are required for the assessment of the concentration-effect relationships. Pharmacokinetic modelling was used to interpolate the individual concentration values for each ECG sampling time. Drug concentrations below LOQ were set to zero.

Given that study analysis was performed in a blinded manner, no prior information on the pharmacokinetics in animals was considered for the purposes of this analysis. Population pharmacokinetic modelling of the drug concentrations in dogs was performed using non-linear mixed effects techniques in NONMEM 7.1.2 (ICON, Maryland, USA). Model-predicted drug concentrations were subsequently used in conjunction with QT interval as input into for the analysis of the pharmacokinetic-pharmacodynamic relationship, as determined by the PKPD model previously developed by our group (Chain et al., 2011).

Pharmacokinetic-pharmacodynamic (PKPD) modelling and extrapolation

Model building was performed in WinBUGS version 1.4, as previously described in Chain and Dubois (Chain & Dubois et al., 2013). The R package R2WinBUGS was used to execute

WinBUGS whilst running a session in R 2.12.8. In brief, the PKPD model comprises three components, which are estimated simultaneously during the fitting procedures: an individual correction factor for RR-interval, an oscillatory function describing the circadian variation of the baseline QTc values and a linear function to capture the concentration-effect relationship. These components are summarised in Equation 1:

$$QT = QT_{C0} \cdot RR^{\alpha} + A \cdot \cos\left(\frac{2\pi}{24}(t-\phi)\right) + slope \cdot C \qquad \text{Equation 1}$$

where QTc_0 [ms] is the individually corrected baseline QTc, RR [sec] is the interval between successive R waves, α is the individual heart rate correction factor, A [ms] is the amplitude of circadian rhythm, t is the clock time, ϕ is the phase, slope [ms/concentration] is the linear concentration-effect relationship, and C is the concentration of drug at the time of QT measurements.

This type of parameterisation discriminates between system- and drug specific properties. Consequently, these parameters can be used to compare drug properties across species without the need for further correction factors (Dubois et al., 2014). Instead, a between-species correlation has been identified for the slope of the pharmacokinetic-pharmacodynamic relationship, which can be used to extrapolated findings from dogs to humans (Dubois et al., 2016). The predicted slope parameter in humans was derived from the mean estimates in dogs as well as from the upper boundary of the 95%-confidence interval, with the objective of accounting for the relatively low precision in the parameter estimates.

Limitations

At the moment the correlation between the slope of the concentration-effect relationship in dogs and humans which we have applied to predict the clinical effects of methadone is based on based primarily on drugs which known hERG ion channel blocking activity (moxifloxacin, sotalol, NCE1,NCE2) and two multi-ion channel blockers (cisapride, carabersat). It has been previously observed that cisapride and to a less extend carabersat do not fully fit into the regression line which describes the interspecies correlation (Dubois et al., 2016). Hence, it can be anticipated that other compounds with a multi ion-channel block potential may not be perfectly described by the correlation. Another point to consider is the possibility of non ion channel related QT prolongation due to autonomic drug effects, such as those observed with

opioids (Fossa, 2008). In these cases, it may not be possible to establish whether there is a pro-arrhythmic potential (Fossa and Zhou, 2010; Garnett et al., 2012). In addition, one should not overestimate the fact that lack of understanding of drug metabolism may represent an important drawback when trying to predict the effect in humans from only *in vivo* safety studies. This point becomes critical interspecies differences are anticipated, such as enantioselective metabolism or formation of a metabolite or another moiety, which contributes to the QT prolongation. This is known to occur for methadone (Garrett et al. 1985).

Perspectives

*Improved evaluation of QT-prolongation in early clinical trials.*The relevance of obtaining clinical data for the evaluation of the safety profile of a compound is illustrated by compounds such as verapamil, which shows clear effects on the hERG current, but does not prolong the QT interval in animals or in humans due to its additional calcium channel blocking properties (Chouabe et al., 1998). In reality, it is highly effective in preventing torsade de pointes (Milberg et al., 2005).
As indicated previously, in the present investigation we have assessed the feasibility of predicting QTc interval prolongation from preclinical animal species to humans and have specifically focused on drugs with known affinity for the hERG channel. However, the translational value of such an approach will depend on the characterisation of drug effects on multiple ion channels, as for example is the case of terfenadine which has both sodium and calcium channel blocking properties. Ultimately, the contribution of each mechanism needs to be integrated in a quantitative manner into a pharmacokinetic-pharmacodynamic model, where ECG, electrophysiological and pharmacokinetic factors can also be accounted for. Interestingly, ongoing revision of the ICH S7B and possible elimination of ICH-E14 requirements point towards. a shift in the focus from evaluating QT prolongation to evaluating pro-arrhythmic activity using a comprehensive *in vitro* pro-arrhythmia assay (CiPA) (Cavero et al., 2014; Sager et al., 2014, Fermini et al 2016). This may represent an advancement but the approach seems to overlook the numerous discrepancies between *in vitro* and *in vivo* findings. In fact, the observed interspecies differences in the disposition of methadone highlight the fact that any attempt to translate non-clinical findings during drug screening process carry uncertainties and consequently affect the specificity and sensitivity of any approach, i.e., there may be false negative and false positive signals as well as biased estimates. In this regard, we envisage an opportunity to overcome some of these issues by further integration of the CiPA concepts in tandem with a PKPD approach.

From a drug development perspective, it should be clear that irrespective of the degree of uncertainty about the liability for QT prolongation, the use of a model-based approach offers the opportunity to better understand the underlying exposure response relationships in the

context of a clinical study protocol. Here, we have evaluated two studies, which are systematically performed before most if not all drugs progress into Phase III. The proposal for such early evaluation has been describe in previous report, where Chain et al. show that FTIH studies can be used as basis for a comprehensive evaluation of QT prolongation in humans. Our simulation exercise confirms the feasibility of combining intensive ECG monitoring during FTIH studies with pharmacokinetic data to assess the probability of drug-induced QTc-prolongation. A key lesson from our simulations is that for compounds with a weak signal, i.e., shallow slope, it may be necessary to consider adaptive protocols, to ensure additional subjects are included at the relevant dose levels. It was also clear that data from repeated dosing is fundamental when metabolites are responsible for QT prolonging effects. Another important observation was the impact of parallel protocol designs in the estimation of the magnitude of QT interval prolongation both in FTIH and the TQT studies. Parameters estimates obtained from parallel designs were less precise, with larger confidence intervals. This is due to the differences in baseline and between-subject variability in QT interval. Irrespective of other statistical considerations, parallel designs render it more difficult to discriminate differences in system vs. drug-specific parameters.

In contrast to the advantages and statistical robustness of the estimation procedures presented by Chain et al., our analysis was focused on the prediction of QT interval prolongation based on the extrapolation of drug-specific parameters from dogs to humans. Clearly, situations in which parameters lack the required precision or carry high degree of uncertainty may lead to biased conclusions about the potential of a molecule to prolong QT interval. This uncertainty as well as the consequences thereof should not be mixed up with the value of FTIH studies for parameter estimation purposes. In conjunction with a model-based approach FITH protocols are sufficiently robust to detect effects as low as 5 ms, with false positive rates in general lower than what is observed in a typical TQT study. Since FTIH trial is a mandatory step in the drug development process, protocols with intensive ECG monitoring can provide an initial assessment of the compound’s safety profile. This can in turn support the strategic decision to proceed or not with development.

References:

Cavero I, Holzgrefe H. CiPA: Ongoing testing, future qualification procedures, and pending issues. J Pharmacol Toxicol Meth. 2015; 76: 27–37.

Chain AS, Krudys KM, Danhof M, Della Pasqua O. Assessing the probability of drug-induced QTc-interval prolongation during clinical drug development. Clin Pharmacol Ther. 2011; 90(6): 867-75

Chain ASY & Dubois VFS, Danhof M, Sturkenboom MCJ, Della Pasqua O. Identifying the translational gap in the evaluation of drug-induced QTc-interval prolongation. Brit J Clin Pharmacol. 2013; 76 (5): 708–24.

Chouabe C, Drici MD, Romey G, Barhanin J, Lazdunski M. HERG and KvLQT1/IsK, the cardiac K+ channels involved in long QT syndromes, are targets for calcium channel blockers. Mol Pharmacol. 1998; 54(4): 695-703.

Dubois VFS, Yu H, Danhof M, Della Pasqua O. Model-based evaluation of drug-induced QT(c) prolongation for compounds in early development. Brit J Clin Pharmacol. 2014; 79 (1): 148–61

Dubois VFS, Smania G, Yu h, Graf R, Chain ASY, Danhof M, Della Pasqua O. Translating QT Interval Prolongation from Conscious Dogs to Humans. Brit J Clinl Pharmacol. 2016; doi: 10.1111/bcp.13123.

Fermini B, Hancox JC, Abi-Gerges B, Bridgland-TaylorM, Chaudhary KW, Colatsky T et al. A new perspective in the field of cardiac safety testing through the comprehensive in vitro proarrhythmia assay paradigm.” J Biomol Screening. 2016; 21 (1): 1–11.

Fossa AA. The impact of varying autonomic states on the dynamic beat-to-beat QT–RR and QT–TQ interval relationships. Brit J Pharmacol. 2008; 154: 1508–15.

Fossa AA, Zhou M. Assessing QT prolongation and electrocardiography restitution using a beat-to-beat method. Cardiol J. 2010; 17 (3): 230–43.

Garnett CE, Zhu H, Malik M, Fossa A, Zhang J, Badilini F, Li J, Darpö B, Sager P, Rodriguez I. Methodologies to characterize the QT/corrected QT interval in the presence of drug-induced heart rate changes or other autonomic effects.” Am Heart J. 2012; 163 (6): 912–30.

Garrett ER, Derendorf H, Mattha AG. Pharmacokinetics of morphine and its surrogates VII: High-performance liquid chromatographic analyses and pharmacokinetics of methadone and its derived metabolites in dogs. J Pharm Sci. 1985; 74 (11): 1203–14.

Milberg P, Reinsch N, Osada N, Wasmer K, Mönnig G, Stypmann J, Breithardt G, Haverkamp W, Eckardt L. Verapamil prevents torsade de pointes by reduction of transmural dispersion of repolarization and suppression of early afterdepolarizations in an intact heart model of LQT3. Basic Res Cardiol. 2005; 100:365-71.

Sager PT,Gintant G, Turner JR, Pettit S, Stockbridge N. Rechanneling the cardiac proarrhythmia safety paradigm: a meeting report from the cardiac safety research consortium. Am Heart J, 2014; 167 (3): 292–300.
